# Supplementary material for: Involvement of Estrogen and Its Receptors in Morphological Changes in the Eyes of the Japanese Eel, Anguilla japonica, in the Process of Artificially-Induced Maturation
Source: Cells. 2019 Apr 3;8(4):310. doi: 10.3390/cells8040310 (PMC6526474; doi:10.3390/cells8040310)
Supplement: Supplementary file 1 [file cells-08-00310-s001.zip › cells-455904 supplementary 4.3/Table S1.pdf]

Table S1. Gonadosomatic index (GSI), oocyte diameter (OD), eye index (EI) changes in the female Japanese eel after intraperitoneal injections of SPE.

	Stage1 (n=8)	Stage2 (n=13)	Stage3 (n=8)	Stage4 (n=6)	Stage5 (n=6)
**GSI** Range (mean ±SEM)	**0.1 – 0.6** (0.18 ±0.07)	**1.4 – 3.0** (1.8 ±0.1)	**6.4 – 17.3** (12.1 ±1.1)	**16.3 – 45.2** (24.8 ±2.0)	**27.6 – 46.2** (40.7 ±2.8)
**OD** Range (mean ±SEM)	**65.7 – 92.5** (78.4 ±3.6)	**155.5 – 197.1** (174.6 ±3.3)	**306.7 – 423.0** (357.5 ±12.6)	**457.0 – 638.7** (522.0 ±27.4)	**975.8 – 1078.6** (892.2 ±83.3)
**EI** Range (mean ±SEM)	**2.41 – 3.98** (3.04 ±0.21)	**3.16 – 4.89** (3.85 ±0.15)	**4.29 – 5.87** (4.99 ±0.18)	**4.02 – 5.10** (4.73 ±0.16)	**6.63 – 7.15** (6.21 ±0.47)
